# Perception to Performance: Linking Cognitive Lapses & Smartphone Cognitive Assessments in the Einstein Aging Study

**DOI:** 10.1093/geroni/igaf122.1890

**Published:** 2025-12-31

**Authors:** Angel Garcia De La Garza, Carol Derby, Cuiling Wang, Jacqueline Mogle, Richard Lipton, Nelson Roque, Mindy Katz, Laura Rabin

**Affiliations:** Albert Einstein College of Medicine, Bronx, New York, United States; Albert Einstein College of Medicine, Bronx, New York, United States; Albert Einstein College of Medicine, Bronx, New York, United States; Clemson University, Clemson, South Carolina, United States; Albert Einstein College of Medicine, Bronx, New York, United States; The Pennsylvania State University, University Park, Pennsylvania, United States; Albert Einstein College of Medicine, Bronx, New York, United States; Brooklyn College and the Graduate Center, The City University of New York, Brooklyn, New York, United States

## Abstract

There is limited literature examining the relationship between subjective cognitive concerns (SCC) and objectively measured cognitive performance. In the Einstein Aging Study, we collected digital diary data on 18 types of cognitive lapses reported daily over 14 days from 297 participants (mean age = 77.54, SD = 4.82; 68% female; 48% Non-Hispanic White, 41% Non-Hispanic Black, 11% Hispanic). Participants completed four mobile cognitive tests assessing processing speed, visual short-term memory binding, spatial working memory, and working memory precision. SCC was analyzed by calculating the average daily number of reported lapses related to memory (12 items; mean = 1.58, SD = 1.48), executive functioning (6 items; mean = 0.84, SD = 1.07), and total cognitive lapses (18 items; mean = 2.42, SD = 2.17), with higher scores indicating greater self-reported cognitive difficulties. We calculated both average daily performance and within-day variability for all cognitive tests. Within-day synchronous associations between SCC and cognition were assessed using linear mixed-effects models, adjusting for age, sex, ethnicity, depression, and education. Findings showed that for each 1 standard deviation decrease in processing speed performance, participants reported 0.09 additional memory lapses (p = 0.013), 0.07 executive functioning lapses (p = 0.005), and 0.14 total lapses (p = 0.003). For each 1 standard deviation decrease in working memory precision, participants reported 0.15 additional memory lapses (p < 0.001), 0.11 executive functioning lapses (p < 0.001), and 0.24 total lapses (p < 0.001). Similar associations were found with within-day cognitive variability, where greater variability in both working memory precision and processing speed corresponded to increased reported daily cognitive lapses.

